# Public health interventions for developing resilience to contagious diseases: a system dynamics approach

**DOI:** 10.1007/s10729-025-09731-9

**Published:** 2025-10-07

**Authors:** Hajar Sadegh Zadeh, Amir Hossein Ansaripoor, Md Hossan Maruf Chowdhury, Ali Haghparast

**Affiliations:** 1 Department of Mathematics and Statistics, University of Melbourne, Melbourne, Australia; 2 Department of Business, Curtin University, Perth, Australia; 3 Department of Business, University of Technology Sydney, Sydney, Australia; 4 Department of Industrial Engineering, Islamic Azad University of Shiraz, Shiraz, Iran

**Keywords:** Contagious disease, Government interventions, SEIR model, Emergency management, System dynamics modelling

## Abstract

Contagious diseases severely impact health systems and economies, with close contact leading to further spread and fatalities. This paper examines the effects of government interventions on controlling such diseases. Key interventions include media isolation of susceptible individuals, effective quarantining of infected persons, and vaccination. A system dynamics approach models the complexities of government interventions in coronary conditions. We used the SEIR (Susceptible, Exposed, Infected, and Recovered) model and developed a new model to address its shortcomings for a new virus. Resilience actions were defined and plotted based on the emergency management cycle phases: Prevention, Preparedness, Response, and Recovery. The model can be applied to any contagious disease worldwide. We calibrated the model using data from sources like the World Health Organization (WHO) and Centers for Disease Control (CDC), and validated it against official and historical data. A sensitivity analysis was conducted based on various resilience strategies: Isolation Rate Slope, Isolation Efficiency, Minimum Isolation Rate, Quarantine Portion, Quarantine Transmission, Vaccination Rate, and Media Rate Slope. The study identifies key conditions for controlling outbreaks: achieving rapid isolation with a minimum rate above 50% and efficiency above 95%, rapid detection and quarantine above 90% with efficiency over 92%, and an optimal contact rate below 0.2, achieved with a media rate slope of 0.005 and vaccination rate above 90%. These measures can control the disease within 455 days or less.

## Introduction

Contagious diseases can rapidly overwhelm healthcare systems, as demonstrated vividly by the COVID-19 pandemic. During outbreaks with escalating infection rates, healthcare providers face acute shortages of critical resources and skilled personnel, while broader economies struggle due to prolonged shutdowns and disruptions in daily activities [1, 2]. To combat these crises, governments implement diverse measures such as large-scale quarantines, mandatory social distancing, and extensive vaccination campaigns aimed at slowing disease transmission and safeguarding public health. However, selecting appropriate strategies and optimal timing for their deployment involves complex decision-making, necessitating a careful balancing act among disease containment, economic stability, and resource limitations.

These multifaceted considerations highlight the importance of utilizing modeling tools that adequately capture how various interventions impact both disease progression and healthcare capacities. Traditional epidemiological approaches, such as the Susceptible–Exposed–Infected–Recovered (SEIR) models, have significantly enhanced our understanding of infection dynamics and epidemic progression timelines [3]. Nonetheless, many existing models tend to simplify policy interventions, overlooking critical real-world variables such as quarantine efficiency, public compliance with isolation measures, the role of media in influencing behavior, and the dynamic nature of vaccination rates [4]. As a consequence, policymakers often face difficulties in harmonizing immediate preventive measures with strategic long-term planning across phases of preparedness, response, and recovery.

To bridge these critical gaps, this paper proposes a system dynamics (SD) model extending the conventional SEIR framework to more realistically incorporate the complexities associated with government interventions. Specifically, our approach integrates essential factors such as mortality, varying quarantine adherence levels, the dynamic isolation of susceptible populations, and evolving vaccination coverage. By explicitly mapping these components onto the Prevention, Preparedness, Response, and Recovery (PPRR) phases, the developed model offers a comprehensive perspective on how policy decisions made at different epidemic stages influence subsequent outcomes in terms of disease spread and healthcare demands.

Although the proposed model primarily targets diseases transmitted directly through airborne or droplet-based routes (such as COVID-19 and influenza), we acknowledge that further adaptations would be necessary for vector-borne diseases like malaria or dengue fever. Vector-borne diseases entail distinct ecological and environmental dynamics, making them fundamentally different from direct human-to-human transmission. Hence, the current assumptions regarding uniform transmission patterns and direct infection routes render this model most applicable to diseases where direct interpersonal contact is the primary transmission mode.

The significance of this study lies in its ability to provide actionable insights for public health authorities and policymakers. Effective modeling can illuminate critical thresholds, such as minimal isolation rates or optimal vaccination timing, that can significantly alter healthcare outcomes. For instance, enforcing strict quarantine procedures or rapidly scaling vaccination efforts could prevent healthcare systems from being overwhelmed. However, each measure carries social, economic, and logistical burdens that must be carefully weighed. By leveraging the robust framework of system dynamics, decision-makers can effectively compare different intervention scenarios, anticipate potential system bottlenecks, and strategically deploy resources for maximum impact.

Accordingly, this research aims specifically:

To explore factors influencing the spread of directly transmitted infectious diseases (e.g., COVID-19, influenza) within a dynamic model incorporating realistic intervention complexities and policy feedback loops.

To identify effective public health strategies—such as isolation, quarantine, and vaccination—that governments can adopt across different phases of the PPRR cycle to reduce pressure on healthcare infrastructure.

The remainder of this paper is structured as follows: the subsequent section reviews pertinent literature on epidemic modeling approaches and governmental public health responses; the following section elaborates on our methodology and detailed model formulation; subsequently, we present and discuss the simulation results along with comprehensive sensitivity analyses; and finally, we conclude with practical implications and suggest potential directions for future research.

## Literature review

Various factors contribute to the spread of infectious diseases like COVID-19, including person-to-person transmission, droplets, aerosols, airborne transmission, and surface transmission [5]. Effective government interventions are crucial in controlling such diseases. This section reviews the literature on: (1) government-led public health interventions in epidemic conditions, (2) the SEIR model in epidemic situations, (3) resilient strategies based on the *PPRR* framework, and (4) system dynamics modeling for pandemics. Throughout, we highlight significant research gaps and clarify how our study addresses them.

### Government interventions for epidemic management

Government roles are vital in managing public health crises. Studies have shown that early prevention, preparedness, and response are essential for effective crisis management [1]. Successful government-led interventions, like those seen in China during COVID-19, have significantly reduced transmission rates [2, 6, 7]. For example, Svoboda et al. (2004) found that public health control measures during the SARS epidemic in Toronto markedly decreased transmission rates in non-controlled environments [6]. Similarly, Chinese researchers illustrated the government’s capacity to control epidemic spread using big data [2]. However, not all interventions succeed; their effectiveness often depends on public acceptance and compliance [8].

Historical examples include British interventions during the Rinderpest outbreak and local government actions during the cholera epidemic in China [7, 9]. Effective measures often require strong collaboration between governments and the public, emphasizing the importance of crisis management capabilities [10, 11]. For instance, Zhang (2015) noted that during the SARS epidemic, citizens’ behavior was subject to more direct government-imposed limitations, requiring significant behavioral changes and compliance [10].

Araiinejad (2020) assessed the impact of local and state government restrictions in Texas, finding significant effects on COVID-19 transmission [4]. Using parameter estimation from the 1918 flu pandemic, Lin Q et al. (2019) developed a conceptual model for COVID-19 in Wuhan, suggesting effective government interventions [12]. Giordano et al. (2020) implemented public health interventions in Italy, highlighting the difference between diagnosed and non-diagnosed infections [13]. Jiayi et al. (2024) emphasize that establishing a targeted mass screening program is key to swiftly identifying infected individuals, isolating them, and thereby controlling transmission [14].

Although these studies underscore the effectiveness of early and coordinated intervention (e.g., quarantining, screening), they often analyze single or isolated measures. Real-world policy enactment involves interdependent decisions—e.g., quarantines plus vaccination campaigns plus media influence—and these interventions shift dynamically as case numbers rise or public behavior changes. Our approach addresses this gap by using system dynamics to capture multi-policy interactions and feedback loops across different stages of an epidemic.

### SEIR model and epidemic situations

The SEIR model has been instrumental in evaluating the effectiveness of control measures during epidemics. It has been used globally to predict epidemic dynamics and assess intervention strategies. For instance, Alberto G. et al. (2020) applied the SEIR model to the COVID-19 outbreak in Italy, comparing intervention efficiencies across different countries [15]. Samuel M. et al. (2020) used a modified SEIR model to demonstrate the necessity of control measures such as social distancing and travel bans to slow COVID-19 spread [16].

Other studies, like Carcione J et al. (2020) and Prem K. et al. (2020), have further developed the SEIR model to include spatial diffusion and contact matrices, respectively, enhancing the model’s applicability to real-world scenarios [17, 18]. Carcione J et al. (2020) used a deterministic SEIR model incorporating spatial diffusion for a more precise two-dimensional estimation of disease spread [17], while Prem K. et al. (2020) performed dynamic analyses using an SEIR model with distinct incidence rates for exposed and infective individuals, integrating location-based physical distancing [18]. Boulaaras S. et al. (2023) focused on estimating the basic reproduction number ($R_0$) to understand outbreak dynamics, validating their model with real-world data to show strong alignment between simulations and observed outcomes [19]. Meanwhile, Tai D. et al. (2021) stressed that widespread vaccination is crucial to ending the pandemic, drawing attention to the practical hurdles in vaccine distribution [20].

While advanced SEIR models provide valuable epidemiological insights, they typically treat policy interventions as static assumptions—for instance, a fixed quarantine rate or contact reduction. In reality, intervention effectiveness may evolve with public compliance, media campaigns, and vaccine uptake. Our study extends SEIR by incorporating dynamic intervention variables, including quarantine adherence, isolation efficiency, and media-driven behavior changes, thus more accurately reflecting real-time policy impacts.

**Fig. 1:**
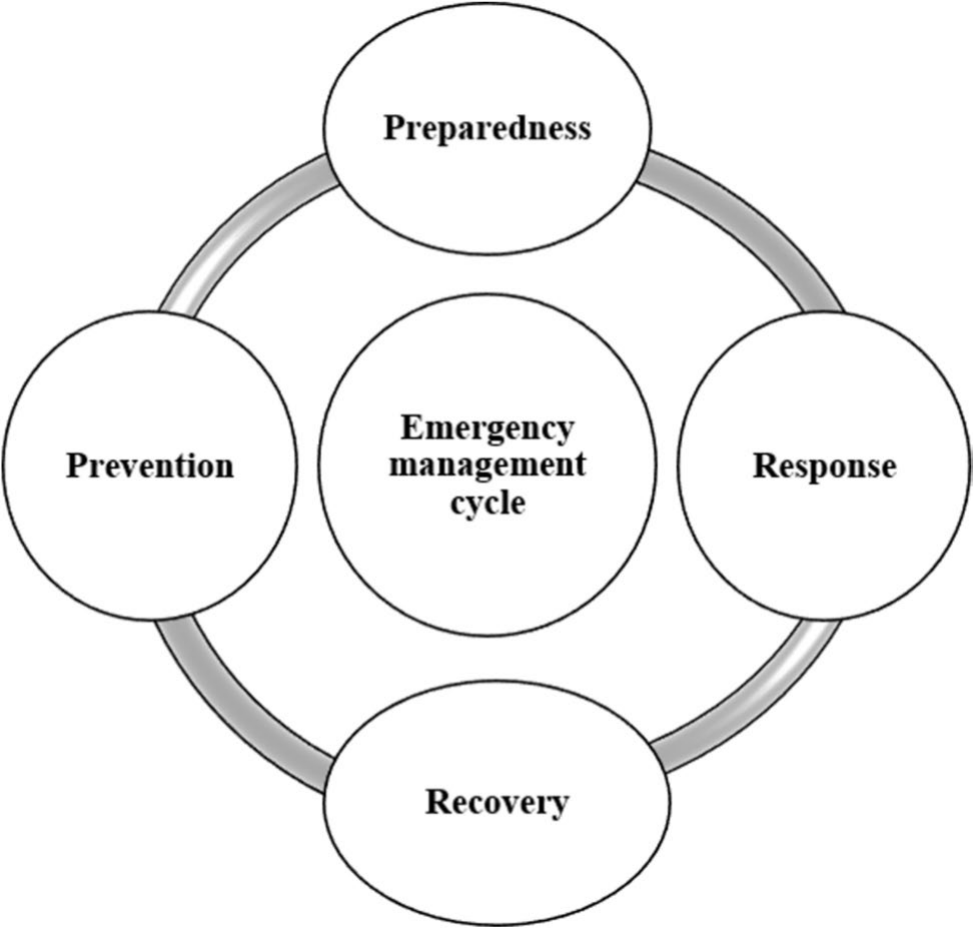
Emergency management cycle

### Resilient strategies based on PPRR in emergency management

The emergency management cycle encompasses *Prevention, Preparedness, Response, and Recovery (PPRR)* [21], guiding strategies to mitigate, handle, and recover from crises (Fig. 1). Resilience involves the system’s ability to return to or improve its state following a disruption [22, 23]. Q.P. Bowman et al. (1999) applied PPRR principles to predict trends in animal health emergency management [24], and Anastasia S. et al. (2021) connected food systems with pandemic response, showing how interdependencies can affect overall crisis outcomes [25]. The WHO’s PPRR framework for measles underscores the importance of timely outbreak identification and consistent immunization efforts [26]. Hrayer A. et al. (2024) further highlight limitations of preventive measures alone and stress the need for robust preparedness and coordinated responses [27].

Although PPRR-based frameworks underscore the value of holistic crisis management, they often lack a quantitative link to epidemiological models. This gap makes it difficult to evaluate how actions in the prevention or preparedness stage affect disease transmission in the response or recovery phase. By embedding PPRR phases into our SEIR-based SD model, we aim to bridge this gap, enabling a more integrated examination of how sequential interventions can collectively improve resilience against infectious disease outbreaks.

### System dynamics modeling and pandemic

System dynamics (SD) modeling has been crucial for understanding and managing contagious diseases, as it incorporates feedback loops and delays in disease transmission and policy effects [11, 28]. Studies in Japan and Singapore used SD modeling to evaluate social distancing policies and test strategies, informing intervention recommendations [29, 30]. Charlle S. et al. (2020) developed an infection transmission framework in Singapore to capture interdependencies among different variables [30]. Additionally, Gabriel R. et al. (2020) created a modeling tool for Chilean officials, enabling them to project infection and death rates under varying social-distancing measures [31]. Sharif S. et al. (2022) extended SEIR to include hospital capacity, medicine supply, and staff functionality, illustrating how multiple non-pharmaceutical interventions (NPIs) shape disease outcomes [32].

Beyond SD, recent optimization-based approaches have sought to determine optimal policy levers. For example, Bertsimas et al. (2021) [33] used data-driven models to prescribe targeted COVID-19 policies; Büyüktahtakın et al. (2018) [34] proposed an epidemics–logistics framework for Ebola response; Giordano et al. (2020) [35] examined nationwide interventions in Italy; and ShamsEddin et al. (2024) [36] studied large-scale testing policies. These methods excel at finding best-case strategies under specific constraints but can underemphasize the dynamic feedback between interventions and evolving disease conditions.

While optimization models offer prescriptive insights, they may not fully capture how adaptive measures can evolve as the outbreak progresses. In contrast, our SD model focuses on scenario analysis and feedback-rich interactions (e.g., how rising infections might trigger stricter quarantines or shift vaccination efforts). By uniting SEIR dynamics with policy feedback within the PPRR framework, we provide a complementary perspective that highlights potential trade-offs between immediate interventions and long-term recovery strategies.

### Summary of the literature gaps

A review of the previous subsections reveals several gaps that this study aims to address. First, existing analyses often focus on single interventions, despite real-world responses involving multiple, interdependent policies. Second, even though advanced SEIR research yields valuable epidemiological insights, policy measures in these models tend to be applied as fixed assumptions, overlooking real-time changes in public compliance and intervention efficacy. Third, while the Prevention, Preparedness, Response, and Recovery (PPRR) framework highlights a holistic approach to crisis management, it is rarely linked quantitatively to epidemiological modeling, thereby obscuring the sequential and cumulative impact of actions taken during each phase. Finally, recent optimization-based models prescribe best-case intervention strategies but often miss the dynamic feedback loop wherein policy effectiveness evolves with the outbreak trajectory.

To bridge these gaps, the present study employs a system dynamics (SD) approach that simultaneously considers multiple policy measures, integrates adaptive intervention variables into SEIR modeling, embeds PPRR principles directly into the epidemiological framework, and complements optimization paradigms by focusing on feedback-rich, scenario-based analyses. Through this integration, the proposed model provides a more comprehensive and adaptable decision-support tool for policymakers confronting rapidly changing epidemic conditions.

## Research methodology and design

A system dynamics (SD) approach is employed to develop the model, with the entire process divided into two main phases: data collection, followed by model development and sensitivity analysis. The details of these two phases are presented in Sections 3.1 and 3.2.

### Data

In the data gathering phase, all data were collected according to the requirements and objectives of the model. Because the disease is still spreading worldwide, to obtain basic model data, we obtained official data such as infection rate, latent period of disease, disease recovery period, and other parameters from three reliable sources: World Health Organization (WHO), Centre for Disease Control (CDC), and European Centre for Disease Prevention and Control (ECDC). In this study, January 30, 2020, is taken as the onset date of the disease, as this is when WHO reported Strange Coronavirus-Related Pneumonia in Wuhan, China [37]. The data gathered for this study covers the period from the onset date until April 30, 2021 (455 days). Due to the uncertainty of the model parameters in different situations and the lack of detailed studies, average values are used. The values for all model parameters can be found in Appendix A, Table 5.

### Model development and sensitivity analysis

After data collection, the second step was to create a new SD model based on the SEIR base model. In this study, SD modeling is used to investigate the complexity and predict systemic behavior with respect to government-led public health interventions in coronary conditions using feedback and scenarios, and to examine the sensitivity of the system to parameters and variables. Using the SEIR base model and addressing its gaps with respect to this new virus, we developed a new model. The base model was modified according to current conditions, needs, possible actions, and interventions.

**Fig. 2:**
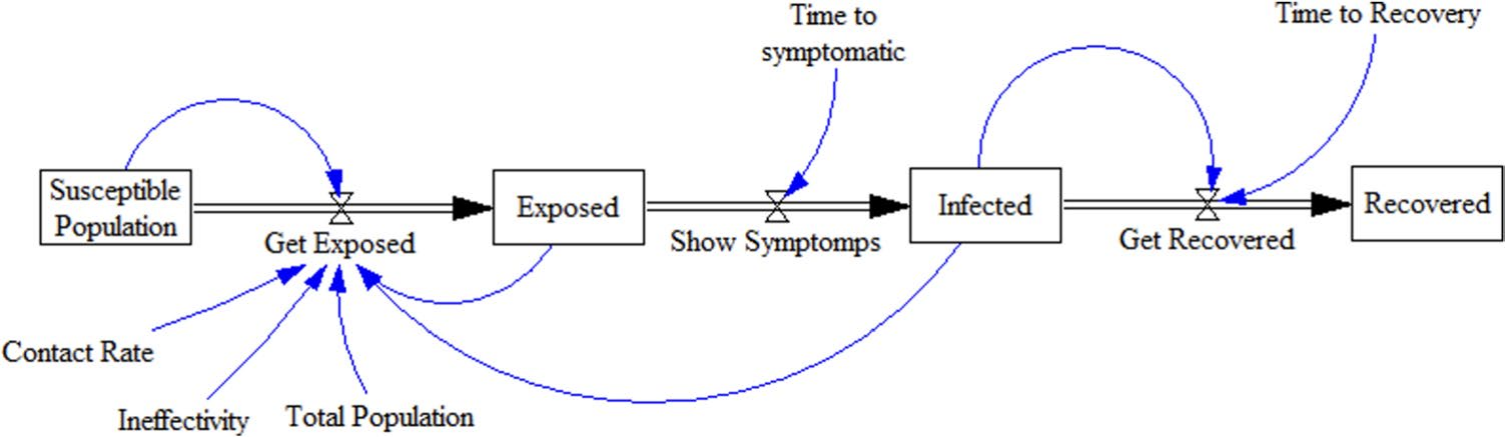
Emergency management cycle and SEIR model

All government-led public health interventions used for controlling and managing COVID-19 are divided into four main categories: prevention, preparedness, response, and recovery, based on a resilient emergency management cycle (Fig. 2). Government actions should be examined through the main measurable stocks (rates of Susceptible, Exposed, Infected, and Recovered individuals). Therefore, we define, extract, and plot the actions of the emergency management cycle (PPRR) phases in relation to each of the main stocks. Table 1 shows a comparison between the emergency management cycle and the original SEIR model presented in Fig. 2.

As shown in Table 1, government-led health intervention comprises four phases as explained below:

- **Prevention:** The goal of government-led health intervention is to reduce the number of people susceptible to contracting the disease by applying a set of measures to prevent contact and isolate healthy people to reduce the risk of disease transmission.
- **Readiness:** The second step is to prepare for people who have been exposed to the disease and are more likely to develop symptoms and illness.
- **Response:** The third measure applies to people who show symptoms and have been infected or who do not show symptoms (asymptomatic).
- **Recovery:** The fourth measure applies to people who should be quarantined in a hospital or at home after the onset of symptoms, and are recovering from the illness.

The base SEIR model (Fig. 2), which includes Susceptible, Exposed, Infected, and Recovered, is incomplete and has been simplified by many assumptions. For instance, the mortality rate, quarantining of the patients, isolation of susceptible individuals, the role of media, and vaccination are not considered in this model. Hence, there is a need to extend this model and propose one that takes into account resilient actions based on an emergency management cycle (PPRR). Therefore, a stock and flow diagram will be developed based on the SEIR model.

Then the proposed stock and flow diagram will be translated to a simulation model using the VENSIM software package. Once the initial data have been gathered, they will be validated to ensure that the model is appropriate. The validation is done according to official data and also the method of Upper and Lower Boundary Conditions. Then, by applying different scenarios, to ensure reliability, sensitivity analysis was conducted on the input parameters. During the sensitivity analysis, target variable values were evaluated by systematically changing input parameters, with each step isolating the effect of a single input variable on a specific stock or outcome.

**Table 1:** The Phases of government-led health intervention

Model	Phase 1	Phase 2	Phase 3	Phase 4
Emergency management Cycle	Prevention	Preparedness	Response	Recovery
SEIR base model	Susceptible	Exposed	Infected	Recovered

## Model development

### Prototype model

The base SEIR model (Fig. 2) was utilized as the prototype model, as it is commonly applied in studies related to the prevalence of disease [38]. In this study, the base model was modified according to current conditions and possible needs and actions. The components of the prototype model are presented in Table 2 below.

**Table 2:** Components of the prototype model

Components	Definitions
Susceptible population	This variable, which is a type of accumulation variable, indicates the population that is susceptible to disease during the period being studied. This population is actually the whole population in the beginning. Over time, as people become exposed or die, the susceptible population decreases. This decline actually begins with exposure. Those who have recovered are added to this population.
Contact Rate	Indicates the normal and unprotected communication rate between people in the community. This rate is initially low (Initial Contact Rate). Although the disease has access to small local populations, over time (Contact Rate Duration), as the community becomes larger, it reaches its maximum with a slope (Contact Rate Duration). On the other hand, this rate (without protection) decreases with the recognition of the disease due to education and healthcare, which is shown in the model developed in the following sections.
Infectivity	This variable (infectivity or infection rate) depends on the nature of the pathogen. Here the virus in question has a very high pathogenicity or contagiousness. This variable indicates the possibility of the disease being transmitted by an infected person to healthy people if normal contact is made between them. It is very difficult to calculate this probability accurately, but good estimates are usually obtained for it.
Total Population	The whole population at the onset of the disease. Here, the population count is taken from the latest official census; estimates are available from the Australian Bureau of Statistics Center (7.9 billion).
Get Exposed	The number of people exposed to the disease daily. In outbreak models, the period is usually days. This rate is based on the percentage of the susceptible population who become infected. To calculate this rate, the variables of contact rate, the ability of the virus to spread or infectivity, and the ratio of infected people to the whole population are used.
Exposed	This variable indicates the accumulated number of people who have been exposed, but those who are infected are reduced by this accumulation.
Show Symptoms	The daily number of people who develop symptoms after being exposed to the virus; that is, they have been diagnosed as having the disease.
Time to Symptomatic	The average time it takes for people to show symptoms. This is the incubation period of the disease.
Infected	The number of patients in each period. This means that those patients who have recovered are not included in this total.
Get Recovered	The number of patients who recover daily. This rate is obtained by dividing the number of patients by the disease period.
Time to Recovery	The average amount of time that an infected person is ill.
Recovered	The total number of patients who have recovered.

Then the proposed stock and flow diagram will be translated to a simulation model using the VENSIM software package. Once the initial data have been gathered, they will be validated to ensure that the model is appropriate. The validation is done according to official data and also the method of Upper and Lower Boundary Conditions. Then, by applying different scenarios, to ensure reliability, sensitivity analysis was conducted on the input parameters. In sensitivity analysis, the values of the target variables were examined by changing the values of the input variables. In each step, the effect of changes on only one stock or target variable was examined.

### Proposed model

**Fig. 3:**
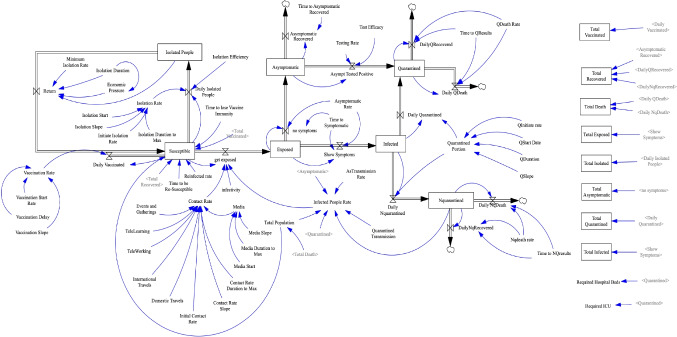
The proposed model with new modules

As seen in Fig. 2, the basic model is concise and has been created by considering many simplified components. However, death is not considered in this model; nor are disease diagnosis and quarantine, population isolation, and the role of media and vaccination. Hence, a new real-world model was developed that includes the above variables (Fig. 3). The new components of the model are described below. In addition, since some of the previous components are slightly different in the new model, those components have been re-introduced based on their new role. The parameters of the model and the values assigned to them based on official data are given in Appendix A. Also, all the defined equations between the parameters and variables of the model are presented in Appendix B. Below, the components of the prototype model are examined by separating the different PPRR phases. (Note that the phases may overlap.)

#### Phase 1: prevention

##### Isolation of healthy susceptible individuals

(Isolation Rate) refers to the separation of part of a healthy population in such a way that any uncontrolled contact with others is eliminated. This is different from reducing the contact rate, as it actually reduces the contact rate to zero for some of the community. Delayed isolation begins at a minimum rate after the onset of the disease (Isolation Start) and gradually increases (Isolation Slope) until it finally reaches a final value over a period of time (Isolation Duration to Max). This separation can last until the final appearance of the disease or it can be shortened (limited separation) to (Isolation Duration) days. In the case of limited separation, the separated individuals return to the community after the separation period at the rate of (Return). This rate depends on the (Economic pressure) parameter. The longer the separation time, the steeper the separation. Additionally, some isolated people may not return until the end of the disease, in which case the rate of the remaining people is determined by (Minimum Isolation Rate). (Isolation efficiency) indicates the success rate of separation as separation may not be completely successful for any reason.

##### Vaccination of healthy susceptible individuals

Vaccines are a modern means of combating COVID-19, and it is tremendously encouraging to see so many vaccines being developed and proving to be effective. Working as rapidly as they can, researchers globally are collaborating to develop, test, and produce vaccines that will save lives and end this pandemic [39]. About a year after the first outbreak of COVID-19, vaccinations began on a global scale. (Vaccination rates) were initially very low and then increased (Vaccination Slope). Vaccination has also been delayed (Vaccination Delay). The daily statistics indicating the number of people who have been vaccinated are obtained by multiplying the vaccination rate by the population of susceptible individuals.

#### Phase 2: readiness

This phase involves preparing people who have been exposed to the disease and are more likely to develop symptoms. It is vital to know the number of people who have been exposed. This number is calculated using the variables of contact rate, infectivity, and the ratio of infected people to the whole community.

##### Limiting person-to-person physical contact

As shown in the base model, contact rate indicates the normal and unprotected communication rate between people in the community, defined by initial contact rate, contact rate slope, and contact rate duration. In reality, government-led health interventions such as media can also influence the contact rate.

##### Promoting media

The impact of media on responding to the disease outbreak and observing health principles is another variable included in the model. The media can affect and reduce the contact rate. This effect usually starts with a delay (Media Start) and gradually increases (Media Slope) until it reaches its maximum after a period of time (Media Duration).

#### Phase 3: response

This phase involves responding to people who show symptoms and those who have been exposed but are asymptomatic and need to be quarantined during the disease’s incubation period.

##### Necessary actions for infected people

The infected people rate represents the number of people who transmit the disease to others. According to the CDC, disease transmission is measured by the ratio of symptomatic patients to the general population. However, studies show that asymptomatic individuals can also spread the disease, potentially as much as 10% [40]. Quarantined patients may also spread the disease (Quarantine Transmission).

##### Separation of asymptomatic and symptomatic people

Symptomatic patients are those who develop symptoms after a latent period (Time to symptomatic). The daily number of symptomatic patients is indicated by the (Show symptoms) rate, typically around 5.1 days [41]. Asymptomatic individuals are those infected with the virus but do not develop detectable symptoms. The proportion of these patients is shown by the (Asymptomatic rate), and their daily number (No symptoms) is separated from the total number of exposed patients after the latent period.

#### Phase 4: recovery

The recovery phase includes actions taken to deal with people who should be quarantined in a hospital or at home after the onset of symptoms and illness (home-quarantined individuals are considered as non-quarantined people).

##### Identification and quarantine of patients

After the onset of symptoms, patients are identified and quarantined. However, not all symptomatic patients are identified. Due to lack of space and equipment, not all identified patients are quarantined in hospitals and medical centers. Many are told to quarantine at home, although some do not comply with quarantine requirements. As a result, only a proportion of patients are quarantined (Quarantine Portion). It may take some time for the disease to be recognized and for quarantine to begin (Quarantine Start). Quarantine starts at an initial rate (Quarantine Initiate Rate) and reaches a maximum with a slope (Quarantine Slope) over time (Duration Quarantine). The number of quarantined patients per day is determined by multiplying this ratio by the total number of patients with symptoms. This flow is shown by (Get Quarantined). By adding all numbers in this flow, the net number of quarantined patients per day is obtained (Quarantined). Other symptomatic people who are not quarantined enter the non-quarantined category (Nquarantined) with the flow of (Out of Quarantined), and are the main spreaders of the disease. However, due to people failing to fully comply with quarantine protocols, or because of human errors of patients or caregivers, both at home and in a hospital, a small percentage of quarantined patients transmit the disease.

It should be noted that the time required to achieve the maximum quarantine of people actually depends on the total population and the time when quarantine began. The later the beginning of quarantine, the greater the number of unidentified infected people, and therefore the longer it takes to identify them. Additionally, the larger the population, the more difficult it is to identify infected people. Therefore, the implementation of a model for different populations with the same time and slope in order to achieve maximum quarantine levels for infected persons is not correct. This should be considered especially for final percentages which are above 90%. Quarantined patients include hospitalized patients and those who have been identified as infected and kept at home by various methods, including general screening. It is assumed that these patients are fully complying with health guidelines.

### Reporting parameters

Reporting parameters are variables that do not affect structural calculations but are included for reporting and quick access to results. Variables such as Domestic Travel, International Travel, TeleWorking, TeleLearning, and Events and Gatherings are added to the model to represent government interventions. Due to the lack of available numeric data, it was not possible to examine the numerical relationship between these variables and the contact rate. However, identifying a close numerical relationship between these factors and the contact level would enhance the simulation’s accuracy. Total stocks, like Total Infected, are added for reporting purposes only. For example, Total Infected differs from Infected as it calculates only the input rate, not accounting for recoveries or deaths.

### Model validity

**Fig. 4:**
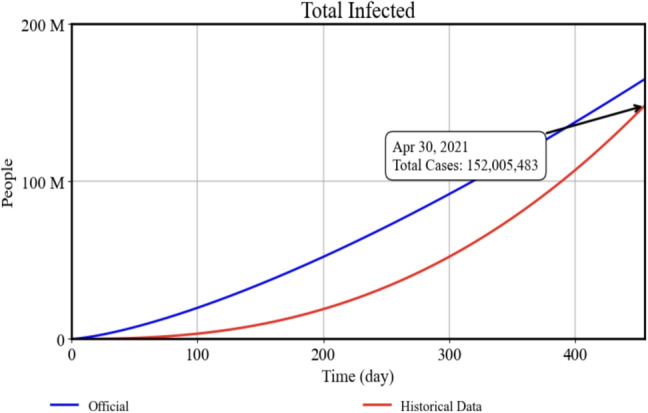
Total Infected: Model results (blue) vs. historical data (red) up to April 30, 2021

We employed two primary methods to validate our model’s structure and outputs. First, we compared the model’s simulated infection trajectory against real historical data. Official global statistics [42] were imported into the VENSIM software, and the daily number of infected individuals was matched against our model’s predictions. As shown in Fig. 4, the model closely follows the historical trend in terms of infection patterns. Specifically, by April 30, 2021 (455 days after the onset of the disease), the number of infected cases stood at approximately 152 million worldwide, while our model estimated nearly 160 million. To quantify the overall fit, we calculated the Root Mean Squared Error (RMSE) between the daily observed and simulated infected cases over the 455-day period and obtained a value of around 3,567,881. In epidemiological models of this scale, an RMSE in the low millions is considered acceptable, reflecting a reasonably close match between the model’s infection curve and real-world data.

**Fig. 5:**
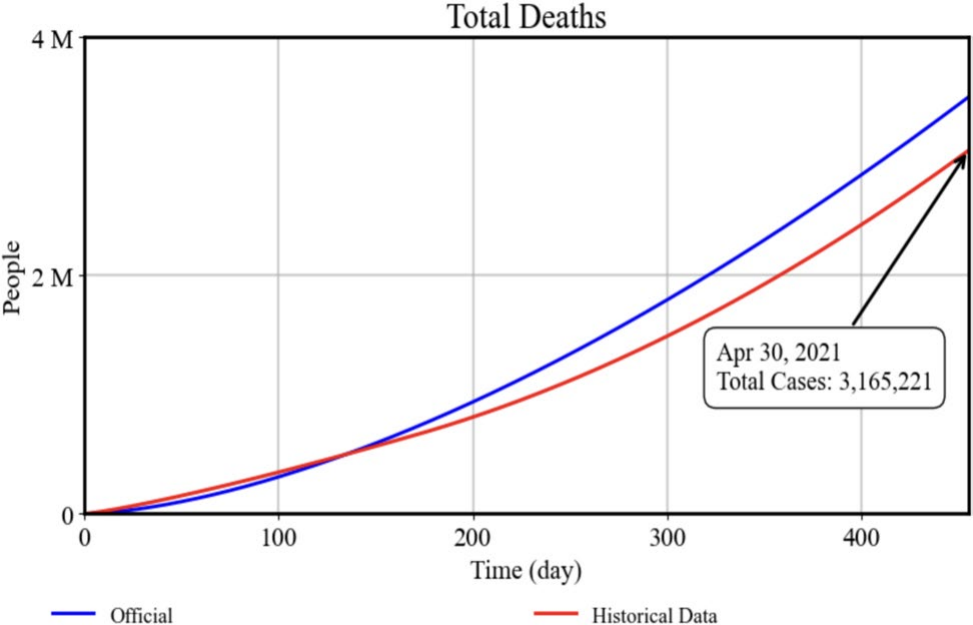
Total Deaths: Model results (blue) vs. historical data (red) up to April 30, 2021

Second, we evaluated the model’s performance in capturing COVID-19–related deaths. According to the World Health Organization (WHO), there were 3,165,221 cumulative deaths globally by April 30, 2021. Our model’s cumulative death estimate for the same period was approximately 3,400,000. To gauge the consistency between observed and simulated mortality, we again calculated the RMSE, which was about 104,708 deaths over the 455-day interval. While global discrepancies in reporting practices can introduce noise into official mortality figures, Fig. 5 demonstrates that our simulated mortality curve aligns reasonably well with the historical record. The RMSE value provides a quantitative measure of the average daily deviation between the model and observed data, indicating that our death projections fall within a plausible range given known uncertainties in international reporting.

**Fig. 6:**
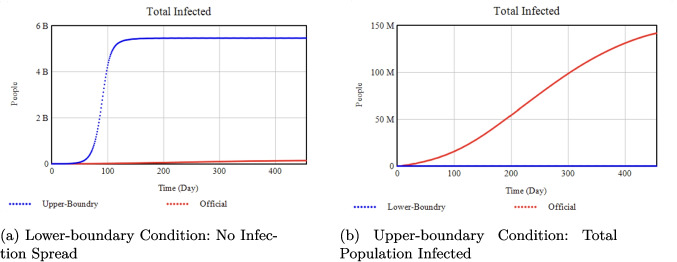
Simulation results under boundary conditions

In addition to these empirical comparisons, we applied boundary testing to further validate the model’s robustness. Under lower-boundary conditions, we assumed maximum control efficacy (i.e., perfect quarantine, isolation, and vaccination rates), resulting in zero disease spread (Fig. 6a). Conversely, in the upper-boundary scenario, where no interventions were implemented, the disease eventually infected the total population (Fig. 6b). Table 3 details the parameter values for each boundary test, both of which produced outcomes consistent with theoretical expectations. Together, these validation steps—empirical data matching and boundary testing—reinforce the credibility of our model and affirm its applicability for policy and scenario analyses.

**Table 3:** The values of variables within the validation scenarios

Parameters	Official	Upper Boundary	Lower Boundary
Isolation Efficiency	0.7	1.0	0.0
Vaccination Slope	$5\times 10^{-5}$	$5\times 10^{-1}$	0.0
Media Slope	0.001	1.0	0.0
Q Initiate Rate	0.375	1.0	0.0
Q Slope	$1.6\times 10^{-7}$	$1.6\times 10^{-1}$	0.0

### Reliability of the model

Parameters in system dynamics (SD) models have inherent uncertainty, making sensitivity analysis crucial for testing the reliability of simulation results. Given that SD modeling is a behavior-oriented simulation approach, it is essential to assess the sensitivity of behavior patterns to understand the impacts of parameter uncertainty on these patterns [43].

**Fig. 7:**
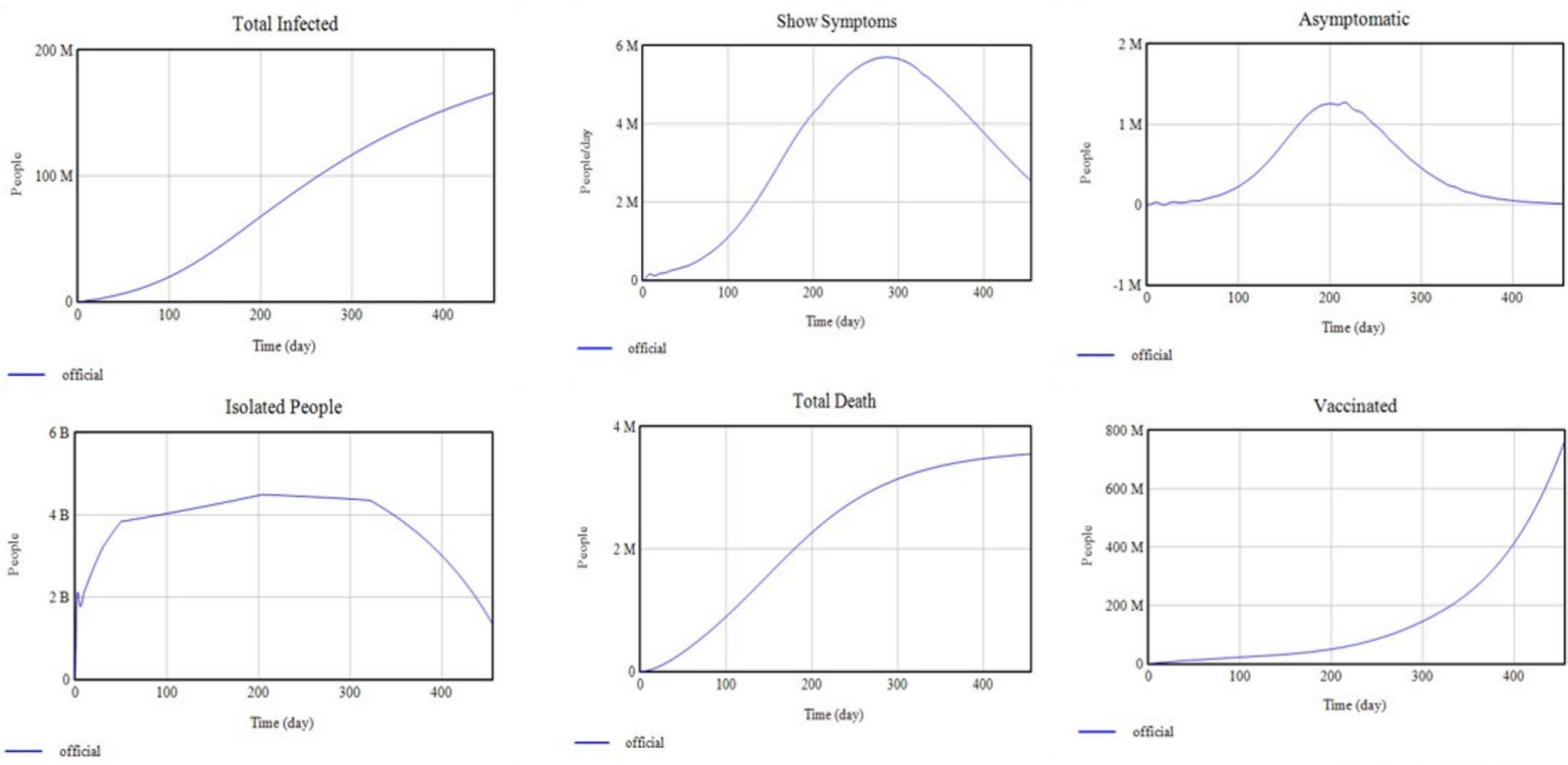
Calibrated model results compared with official data over 455 days from the onset of the disease (January 30, 2020) to April 30, 2021

**Fig. 8:**
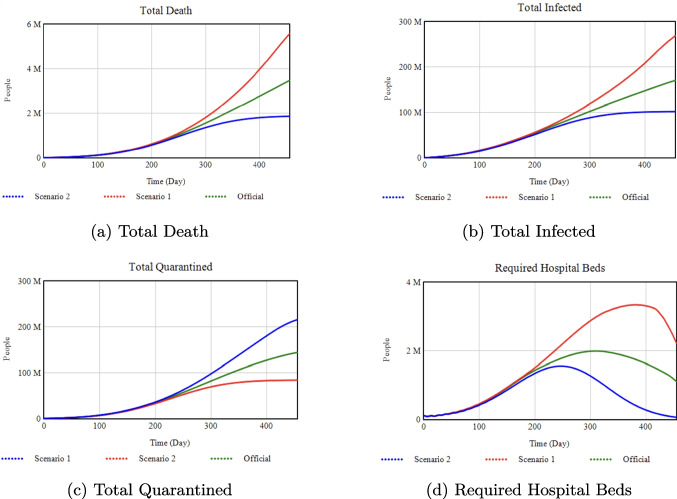
Sensitivity analysis with respect to the Isolation Rate Slope

**Fig. 9:**
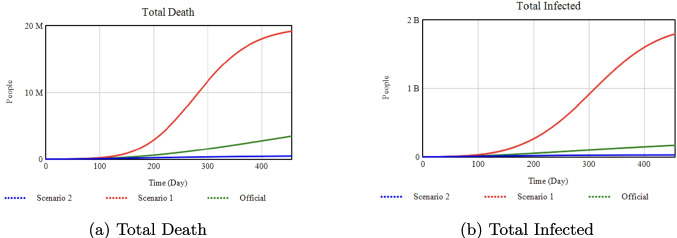
Sensitivity analysis with respect to Isolation Efficiency.

**Fig. 10:**
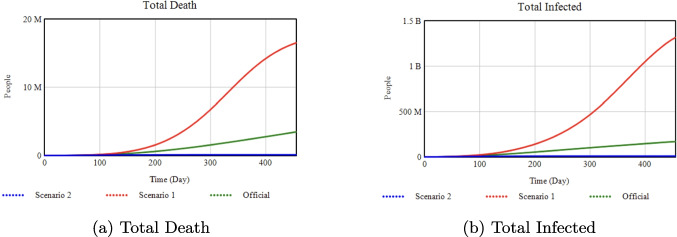
Sensitivity analysis with respect to the Minimum Isolation Rate

**Fig. 11:**
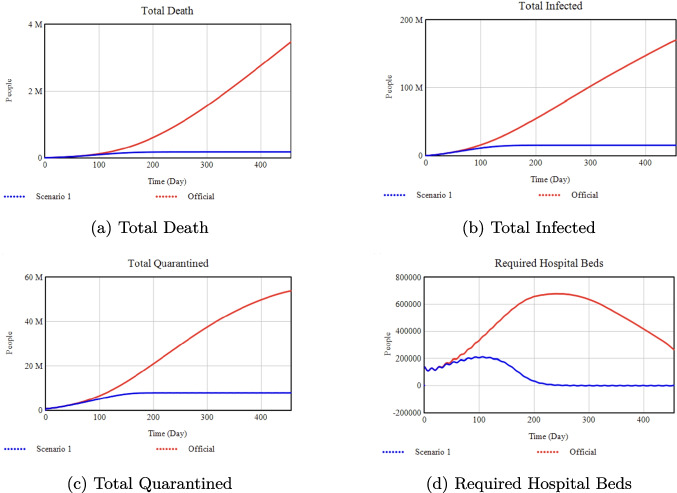
Sensitivity analysis with respect to the Vaccination Rate

**Fig. 12:**
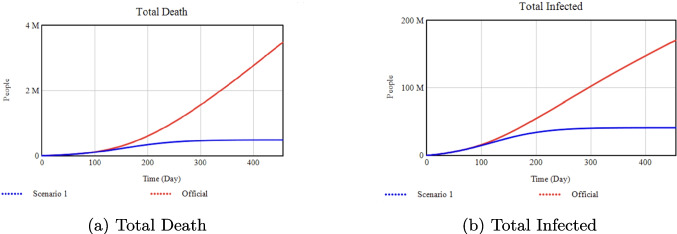
Sensitivity analysis with respect to the Quarantine Portion

**Fig. 13:**
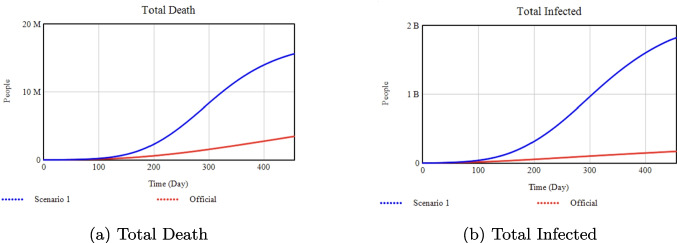
Sensitivity analysis with respect to Quarantine Transmission.

**Fig. 14:**
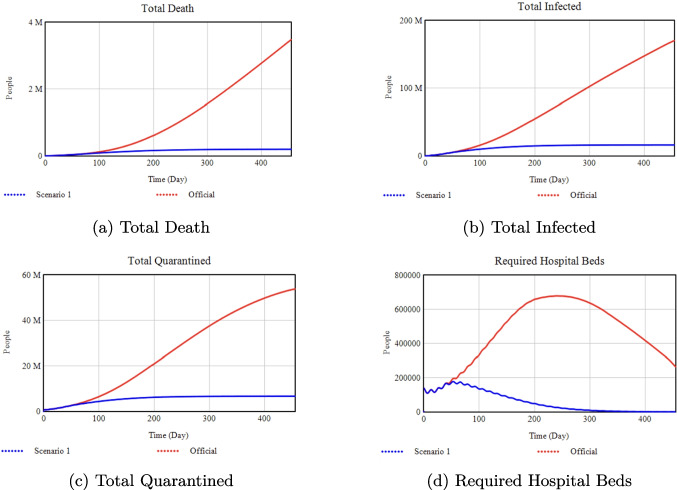
Sensitivity analysis with respect to the Media Rate Slope

In this section, we perform sensitivity analyses on various parameters to ensure the reliability and accuracy of our model. For instance, by reducing a parameter, we examine whether the model output changes as expected. The sensitivity analyses, as shown in Figs. 7, 8, 9, 10, 11, 12, 13, and 14, confirm that our proposed model is reliable and produces consistent results.

## Results

Figure 7 illustrates the simulated trajectories for key model variables (Total Infected, Show Symptoms, Asymptomatic, Isolated People, Total Death, and Vaccinated) compared with official data up to April 30, 2021.

Figures 8–14 present sensitivity analyses for the following parameters: Isolation Rate Slope, Isolation Efficiency, Minimum Isolation Rate, Quarantine Portion, Quarantine Transmission, Vaccination Rate, and Media Rate Slope.

To explore the model’s full range of behavior, we identified two sets of values (Scenario 1 and Scenario 2) that represent *upper/lower* or *extreme* bounds for each parameter. Although such extremes may exceed typical real-world practices, they highlight how the system behaves when an intervention is pushed to its limits. For instance, a very high vaccination rate (0.9) approximates a best-case scenario assuming unlimited resources and logistics, while a much lower rate (0.002) represents a more constrained outcome. In future work, time-varying or resource-limited interventions could better reflect real-world feasibility. Here, our aim is to identify how much each intervention lever can influence key outputs such as total deaths and peak hospitalizations.

### Effect of isolation rate slope on key health outcomes

**Official:** 0.0125, **Scenario 1:** 0.0100, **Scenario 2:** 0.9

*Percentage Changes:* Increasing the Isolation Rate Slope from 0.0125 (Official) to 0.9 (Scenario 2) reduces total infections by about 35% and deaths by 42% by Day 455. Conversely, lowering it to 0.0100 (Scenario 1) raises deaths by 83% above the Official scenario.

### Effect of isolation efficiency on deaths and infections

**Official:** 0.70, **Scenario 1:** 0.45, **Scenario 2:** 0.95

*Percentage Changes:* Raising Isolation Efficiency from 0.70 (Official) to 0.95 (Scenario 2) lowers total infections by approximately 99% and total deaths by 95%. If it drops to 0.45 (Scenario 1), total infections increase by 300% and deaths by 500% compared to Official.

### Effect of minimum isolation rate on deaths and infections

**Official:** 0.20, **Scenario 1:** 0.0, **Scenario 2:** 0.5

*Percentage Changes:* Increasing the Minimum Isolation Rate from 0.20 (Official) to 0.50 (Scenario 2) drastically reduces total infections and deaths by about 99% each. By contrast, reducing it to 0.0 (Scenario 1) drives infections up by 800% and deaths up by 500% compared to Official.

### Effect of vaccination rate on health outcomes

**Official:** 0.002, **Scenario 1:** 0.9

*Percentage Changes:* Switching from a maximum vaccination rate of 0.002 (Official) to 0.9 (Scenario 1) yields a 93% reduction in total infections and a 98% reduction in deaths by Day 455. Quarantined numbers and required hospital beds also drop substantially, by 85% and 100%, respectively.

### Effect of quarantine portion on deaths and infections

**Official:** 0.375, **Scenario 1:** 0.9

*Percentage Changes:* Raising the quarantine portion from 0.375 (Official) to 0.90 (Scenario 1) reduces total infections by 79% and deaths by 88% by Day 455.

### Effect of quarantine transmission on deaths and infections

**Official:** 0.081, **Scenario 1:** 0.5

*Percentage Changes:* Increasing quarantine transmission from 0.081 (Official) to 0.5 (Scenario 1) triggers a major surge: total infections jump by about 1000% and deaths by 500% compared to Official.

### Effect of media rate slope on health outcomes

**Official:** 0.001, **Scenario 1:** 0.005

*Percentage Changes:* Raising the Media Rate Slope from 0.001 (Official) to 0.005 (Scenario 1) lowers total infections by 92% and deaths by 97%. It also reduces quarantined individuals by 87% and hospital beds to nearly zero.

### Combined feasible interventions scenario

The prior subsections examined each parameter independently under extreme assumptions, but real-world policymaking typically involves multiple interventions at moderate levels. Here, we propose a combined scenario with mid-range, feasible values for each parameter, as summarized in Table 4.

**Table 4:** Parameter values in the combined feasible-interventions scenario

Parameter	Official	Feasible Scenario
Isolation Rate Slope	0.0125	0.05
Isolation Efficiency	0.70	0.80
Minimum Isolation Rate	0.20	0.30
Quarantine Portion	0.375	0.70
Quarantine Transmission	0.081	0.30
Max Vaccination Rate	0.002	0.50
Media Rate Slope	0.001	0.003
Testing Rate	0.001	0.004

**Fig. 15:**
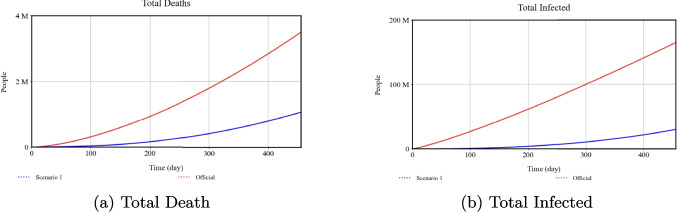
Outcomes under combined feasible interventions vs. Official

We then ran the model from Day0 to Day455 with these simultaneous interventions. Figure 15 compares the Official scenario (red) with the new “Feasible Scenario” (blue). This analysis shows an approximate 83% reduction in total infections and a 72% reduction in total deaths compared to the Official scenario. Although these improvements are less dramatic than in some extreme scenarios, they illustrate how orchestrating moderate interventions can still substantially mitigate the outbreak.

As shown in Figs. 8–10, decreasing the Isolation Rate Slope or Efficiency increases overall disease burden, whereas raising Minimum Isolation Rate dramatically lowers it. Figures 11–14 confirm that higher Vaccination and Media Rates reduce total infections and deaths significantly, while Quarantine Portion and Transmission show similar powerful effects. In sum, our model highlights the critical role that concerted, multifaceted interventions play in controlling COVID-19.

### Robustness of the results

Recalculating results under various assumptions allows us to test the model’s stability and assess how parameter uncertainty affects the outcomes. Our sensitivity analyses demonstrate that the proposed model is robust and reliably captures key trends over a broad range of plausible interventions.

## Discussion and conclusion

According to the findings, the COVID-19 virus can spread rapidly and widely, particularly when there is ineffective isolation of susceptible individuals and inadequate quarantine measures. This underscores the need for more effective public health interventions, which are discussed below.

### Effective government-led public health interventions

Several fundamental measures are essential to control the transmission of COVID-19. Isolating susceptible individuals to minimize contact with infected people is crucial; thus, ensuring maximum isolation efficiency must be a top priority. The more isolated individuals are, the more they can avoid illness and possible death. Another critical measure is the high rate and timely vaccination of susceptible individuals, which can significantly curb the spread of the disease.

Additionally, the identification and quarantine of patients is a vital tool for controlling the disease. Rapid and accurate diagnostic testing is necessary to identify those needing quarantine; achieving a quarantine rate above 90% can lead to significant disease control. It’s crucial to maintain high quarantine efficiency, ensuring that at least 92% of quarantined patients do not transmit the disease to others. Furthermore, keeping the maximum quarantine rate stable is essential, as any decrease could lead to a resurgence of the disease. Governments should provide facilities to quarantine more individuals in hospitals and health centers under strict health protocols.

Maintaining a low contact rate within the community is also vital. The findings suggest that the main cause of successive waves of the disease is the increase in contact rates after restrictions are lifted and initial health guidelines are relaxed. Therefore, investing in public health education and training is necessary. Timely and widespread vaccination is another key policy, as it can significantly reduce mortality and infection rates. A vaccination rate above 90% can almost eliminate mortality.

### Practical feasibility considerations

Despite the clear benefits of high thresholds (e.g., isolation efficiency above 95%, quarantine detection rate above 90%), achieving such levels of compliance presents significant practical challenges. Social, economic, and logistical constraints—especially in resource-limited settings—may limit mass testing, delay quarantining, or reduce the willingness of individuals to isolate for extended periods. Likewise, sustaining high vaccination coverage requires dependable vaccine supply chains and robust public-health campaigns to overcome vaccine hesitancy. Future research could explore smaller-scale implementations of our model in specific cities or regions, allowing parameter calibrations to reflect local healthcare capacities, cultural attitudes, and resource availability. Such context-specific studies would clarify how close real-world interventions can get to these ideal targets and which policy levers (e.g., financial support, ongoing media campaigns, improved detection tools) most effectively boost feasibility.

These measures, if implemented effectively, will reduce the need for extensive isolation and business closures, which would otherwise impose significant economic pressures. However, if these conditions cannot be met, isolating the susceptible population can be an effective alternative. This strategy allows governments and healthcare systems to slow the spread of the disease and optimize detection and quarantine efforts. Adequate patient quarantine and quarantine efficiency will prevent the uncontrolled re-emergence of the disease even as the isolated population is gradually released.

Nevertheless, there is always a risk of a resurgence if patient identification and quarantine are inconsistent. Isolation provides the opportunity to achieve the desired detection and quarantine rates, and in the event of a re-emergence, isolating the susceptible population can help maintain control. If the conditions outlined above are met and maintained, the rate of infection can be curbed shortly after the disease emerges. Otherwise, the disease is likely to re-emerge due to inadequate identification and quarantine or increased contact rates.

In summary, the most effective strategies for controlling the disease are rapid isolation with a minimum rate above 50% and efficiency above 95%, rapid detection and quarantine above 90% with efficiency over 92%, and maintaining an optimal contact rate near 0.2. A media rate slope of 0.005 and a vaccination rate above 90% can collectively control and curb the disease within 455 days or less.

### Real-world evidence supporting key intervention thresholds

While our model identifies certain thresholds for quarantine and vaccination (e.g., a quarantine detection rate above 90% or a vaccination coverage exceeding 90%), it is important to consider whether these figures are consistent with existing real-world examples. Several nations have, in practice, implemented interventions whose outcomes align—at least partly—with the thresholds predicted by our simulations:

- **High Vaccination Coverage:** Countries such as Portugal, Iceland, and the United Arab Emirates exceeded 85–90% full vaccination rates during peak COVID-19 campaigns [44]. Observational data indicate that they subsequently experienced lower hospitalization and mortality rates, which supports our model’s assertion that high vaccination coverage can significantly reduce death and infection trajectories [45].
- **Stringent Quarantine Practices:** Certain regions in Asia (e.g., parts of China, Singapore, and South Korea) implemented rigorous test-trace-isolate strategies with rapid quarantine measures exceeding 80–90% detection rates in initial outbreaks [46]. While exact compliance rates varied over time, their relatively fast identification and isolation of infected individuals contributed to shorter and less severe epidemic waves—again echoing our model’s predictions about the importance of quarantine efficiency.
- **Combined Measures:** Real-world experiences during the COVID-19 pandemic suggest that layers of interventions (e.g., contact tracing, travel restrictions, media awareness, and vaccination) were necessary to keep outbreaks under control. These real-case scenarios underscore the interplay of multiple strategies in reaching effective overall thresholds, in line with our system dynamics approach that highlights the synergy between isolation, quarantine, and vaccination efforts.

Although no single country precisely mirrored our model’s target thresholds (e.g., consistently above 95% isolation efficiency), historical instances demonstrate that the closer interventions come to these levels, the more effectively outbreaks are contained. These real-world findings thus add empirical support to our model’s thresholds, even if practical and sociopolitical constraints sometimes limit implementation. Further case studies focusing on specific localities (e.g., cities or states) could yield additional comparative evidence, helping policymakers gauge how to adjust intervention intensities to match regional capabilities and cultural factors.

### Study limitations

We made every effort to include all the necessary variables and details in the model to reflect reality accurately. However, certain variables, such as Preventing Domestic and International Travels, Teleworking and Telelearning, and Preventing Events and Gatherings, were intended to be added to the model to represent government interventions. Unfortunately, it was not possible to examine the numerical relationship between these variables and the contact rate due to a lack of data. These variables could be incorporated into the model if numerical data becomes available.

In general, restricting domestic travel by limiting access to or closing roads, banning international travel by preventing incoming and outgoing flights and closing borders, shifting from face-to-face work and education to online platforms, and discouraging events and gatherings through fines and venue closures, could effectively reduce the contact rate.

## Data Availability

The datasets used in this study are publicly available and can be accessed through the following links: $\bullet$ **World Health Organization**: COVID-19 Vaccines. Available online: https://www.who.int/news-room/commentaries/detail/transmission-of-sars-cov-2-implications-for-infection-prevention-precautions. $\bullet$ **Healthline**: Reinfections Occur, but Most People Are Protected. Available online: https://www.healthline.com/health-news/covid-19-pandemic-what-we-know-about-coronavirus-reinfections . $\bullet$ **StatPearls**: H1N1 Influenza (Swine Flu). Available online: https://www.ncbi.nlm.nih.gov/books/NBK513241 . $\bullet$ **Emerging Infectious Diseases**: COVID-19: Study Estimates Rate of Silent Transmission. Available online: https://wwwnc.cdc.gov/eid/ . $\bullet$ **Johns Hopkins University Bloomberg School of Public Health**: New Study on COVID-19 Estimates 5.1 Days for Incubation Period. Available online: https://www.sciencedaily.com/releases/2020/03/200310164744.html. $\bullet$ **Times of India**: Mild-Asymptomatic COVID-19 Patients May Not Be Infectious for More Than 10 Days: Study. Available online: https://timesofindia.indiatimes.com/life-style/health-fitness/health-news/mild-asymptomatic-covid-19-patients-may-not-be-infectious-for-more-than-10-days-study/articleshow/78789770.cms. $\bullet$ **Centers for Disease Control and Prevention (CDC)**: COVID-19 Case Surveillance Public Use Data. Available online: https://data.cdc.gov/Case-Surveillance/COVID-19-Case-Surveillance-Public-Use-Data/vbim-akqf/data. $\bullet$ **The Guardian**: Less Than 20% of People in England Self-Isolate Fully, Sage Says. Available online: https://www.theguardian.com/world/2020/sep/11/less-than-20-of-people-in-england-self-isolate-fully-sage-says. $\bullet$ **Wikipedia**: COVID-19 Vaccine History. Available online: https://en.wikipedia.org/wiki/COVID-19-vaccine-History. $\bullet$ **Worldometer**: Covid-19 Coronavirus Pandemic. Available online: https://www.worldometers.info/coronavirus/.

## Appendix A: Table of inputs

**Table 5:** Nomenclature

Parameter	Amount	Unit	Source	Further details
Infectivity	0.55	Dimensionless	[47]	According to the Talha N. et al. [47] model, the infectivity rate for the H1N1 virus is 0.42. This rate could be the lower limit for the coronavirus, which is more pathogenic than the influenza virus, but the exact number is unknown. Accordingly, the rate can be adjusted for the COVID-19 model by increasing the value to 0.55.
Quarantine Transmission	0.081	Dimensionless	[48]	Four individual studies from Brunei, Guangzhou China, Taiwan China and the Republic of Korea found that between 0% and 2.2% of infected people transmitted the virus.
AsTransmission rate	0.1	Dimensionless	[40]	Studies show that a percentage of patients who do not have symptoms may also spread the disease. This can be up to 10% of these patients [40].
Testing Rate	0.001	1/day	[49]	Fraction tested per day based on reference [49]
Test Efficacy	0.90	Dimensionless	[50]	Probability an infected test yields a positive based on reference [50]
Time to Symptomatic	5.1	Day	[41]	According to a study by Johns Hopkins University, this time is 5.1 days [41]. Study shows that mild-asymptomatic COVID-19 patients may not be infectious for more than 10 days.
Time to Asymptomatic	10	Day	[51]	Mild-asymptomatic COVID-19 patients may not be infectious for more than 10 days [51].
Contact Rate	0.2 (On average)	1/day	[52]	Contact rate based on various interactions and social behaviors.
QDeathRate	1/1271=0.0007	1/day	[53]	Based on the analysis of COVID-19 cases.
NqdeathRate	0.014	1/day	[54]	Statistics obtained from 37 countries suggest that the mortality rate is about 1.4% [54].
Time to QResults and NQResults	14	Day	[54]	According to COVID-19 timeline, the average recovery or death time is 14 days.
Isolation start	Day 0=30 January 2020	Day	[55]	On January 30, 2020, the WHO declared the COVID-19 outbreak a global health emergency.
Qslope	1.6e-07	1/(day*day)	[56]	Daily, 1264 people in the world are quarantined in hospital due to COVID-19, so 1264/7.9e+09=1.6e-07.
Isolation Duration to Max	72	Day	[57]	Approximately after 70 days from the onset of the disease, Italy isolated its entire population, so we extended it to the whole world.
Isolation Slope	0.0125	1/(day*day)	[58]	It is inferred that after 70 days, 7.9 billion people were isolated. Hence, approximately 100,000,000 people were isolated daily, which is almost 0.0125 per day.
Isolation Efficiency	0.7	Dimensionless	[58]	-
Minimum isolation Rate	0.2	1/day	[59]	Reference [59] just mentioned England, but we have extended it to the entire world.
Isolation Duration	47	Day	[41]	Isolation extended from 30 Jan 2020 to 30 Apr 2021.
Vaccination delay	320	Day	[60]	A limited vaccination rate started from 24 June 2020, but global vaccination started on 20 Dec 2020, 320 days after the onset of the disease.
Reinfected rate	0.01	Dimensionless	[61]	Research in the United Kingdom found that of the people who had already contracted the SARS-CoV-2 infection, less than 1 percent contracted it again.
Re-susceptibility period	120	Day	[61]	-
Time to Lose VaccineImmunity	180	Day	[62]	Although precise durability of vaccine-conferred immunity can vary, recent analyses suggest a marked reduction in protection around six months [62]. Consequently, we set our baseline *Time to Lose VaccineImmunity* at 180 days to reflect an approximate window for waning vaccine efficacy.
Required Hospital Beds	0.2*Quarantined	Bed	[40]	Studies show that just near to 20% of quarantined population are hospitalized and use hospital beds.
Required ICU	0.05*Quarantined	Bed	[40]	Among people who must be quarantined due to COVID-19, only 5% need ICU beds.
